# Shetti, a simple tool to parse, manipulate and search large datasets of sequences

**DOI:** 10.1099/mgen.0.000035

**Published:** 2015-11-06

**Authors:** Haitham Sobhy

**Affiliations:** Dalian Institute of Chemical Physics, CAS, Dalian, PR China

**Keywords:** comparative genomics, protein/gene sequences, functional motif/domain, consensus pattern, sequence manipulation

## Abstract

Parsing and manipulating long and/or multiple protein or gene sequences can be a challenging process for experimental biologists and microbiologists lacking prior knowledge of bioinformatics and programming. Here we present a simple, easy, user-friendly and versatile tool to parse, manipulate and search within large datasets of long and multiple protein or gene sequences. The Shetti tool can be used to search for a sequence, species, protein/gene or pattern/motif. Moreover, it can also be used to construct a universal consensus or molecular signatures for proteins based on their physical characteristics. Shetti is an efficient and fast tool that can deal with large sets of long sequences efficiently. Shetti parses UniProt Knowledgebase and NCBI GenBank flat files and visualizes them as a table.

## Data Summary

The software and documentation are freely available for research use at https://sourceforge.net/projects/shetti.

## Impact Statement

Shetti is a novel and simple tool created for experimental biologists to analyse, search or manipulate large datasets of sequences efficiently, without the need to write additional scripts or codes.

## Introduction

With the increasing number of newly isolated species and genome sequences in recent years (Benson *et al.*, 2013), the need for bioinformatics tools has grown. One of the critical challenges is manipulating, editing and processing huge numbers of gene or protein sequences. Manipulating long and multiple protein or gene sequences, for example hundreds of sequences with more than 10 000 nt or amino acid residues, can be a complicated task for biologists with inadequate knowledge of programming or bioinformatics tools. BioEdit (http://www.mbio.ncsu.edu/bioedit/bioedit.html) and Unipro UGENE (Okonechnikov *et al.*, 2012) are free sequence visualization and manipulation tools. The BioWord tool was developed to manage DNA and protein sequences within Microsoft Word processing software (Anzaldi *et al.*, 2012). DAMBE was developed for phylogenetic analysis purposes, but the tool contains other modules for sequence manipulation (Xia, 2013). These tools can create a DNA consensus, design primers, translate DNA to proteins, generate consensus logos, and reverse-complement a sequence. They can be linked to third-party applications (such as sequence alignment and molecular phylogenetics tools). Nevertheless, these tools cannot be used to search for data in large datasets. By contrast, the BlockLogo tool is designed for visualizing consensus motifs (Olsen *et al.*, 2013), whereas the Minimotif Miner database is designed for finding motifs within a sequence (Mi *et al.*, 2012). These tools do not support browsing, manipulating or searching large-scale omics data. Their shortcomings also include parsing and manipulating large and raw data in GenBank or UniProt files. These issues are challenging for experimental biologists without knowledge of bioinformatics.

To overcome these shortcomings, we have developed Shetti to mine, browse, manipulate and search large datasets of large sequences. The word ‘Shetti’ means digging out or mining in ancient Egyptian, and this reflects the main purpose of the tool. Shetti digs out or mines for useful information from hundreds of sequences. It has a simple and user-friendly interface to retrieve information from plain datasets, and convert raw data files to human-readable format. Therefore, fasta files, and flat GenBank and UniProt files can be browsed and organized easily within tables. Searching for specific species or proteins/genes can also be achieved easily. Shetti searches for specific pattern(s) within multiple sequences, which cannot be achieved by other tools. These options could help to search for particular functional motif(s) within hundreds of sequences, as well as finding the universal consensus, molecular signature or pattern (based on the physical properties of residues) shared among sequences or species within genera.

## Theory and Implementation

**Fig. 1.**
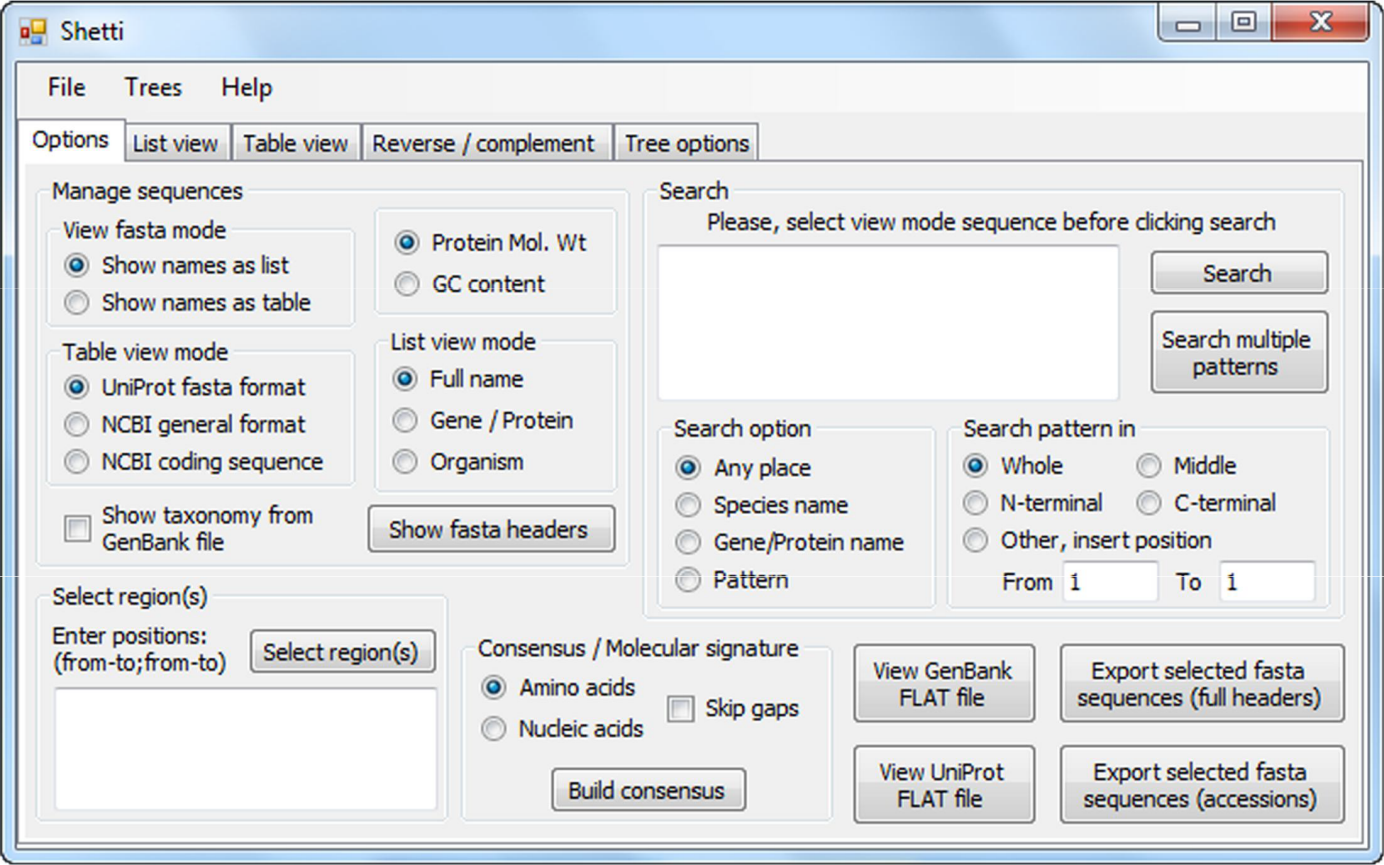
A screenshot of the Shetti interface.

**Fig. 2.**
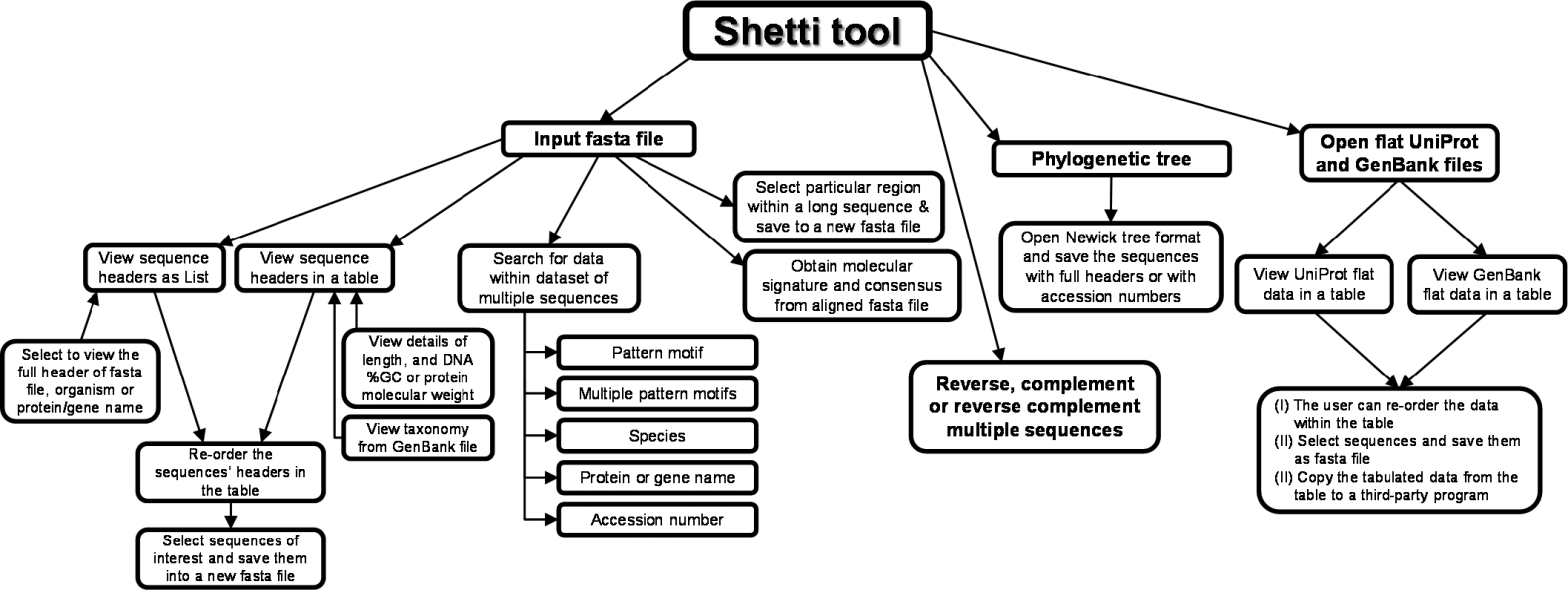
A flow chart of Shetti features.

Shetti has a user-friendly interface (Fig. 1, Figs S1–S4, available in the online Supplementary Material) that offers interactive features to extract information from multiple long sequences (Fig. 2). Shetti accepts fasta files of multiple sequences, nucleic acid or amino acid, as input (for sequence format, see Fig. S5). The tool reads fasta headers and sequences to memory. The fasta headers can be visualized as a list or table of headers. In the list view mode, the full fasta header, protein or gene names, or the species encoding the sequences are listed with check-boxes to choose particular sequences (Fig. S1). In the table view mode, the fasta headers are presented in table columns, which include accession numbers, protein or gene names, organisms, sequence length, nucleotide G+C content or protein molecular mass (Table S1, Fig. S2). The header(s) of interest can be selected and the sequence(s) saved into a new fasta file. Note that regardless of the selected visualization mode, the sequences are manipulated in the same manner.

Moreover, the tool parses UniProt Knowledgebase and NCBI GenBank flat files and visualizes them as a table, which includes accession numbers, gene/protein name, species, organelle, host, taxonomy, length and molecular mass. Table data can be exported as a fasta file, or copied and transferred to a spreadsheet software program. Extracting taxonomy details of species from GenBank files could be helpful for studying protein homology between organisms.

One of the characteristic options in Shetti when compared with other tools is its ability to search multiple sequences. Users can search for particular species names (binominal nomenclature) or even the name of a protein or gene within multiple sequences. Moreover, searching for single or multiple protein patterns (sequence motifs) can be easily achieved using Shetti. Users can choose the search location, such as C-terminal, N-terminal or the entire sequence(s). Sequences containing motifs are saved into a new fasta file. The method of searching patterns follows ExPASy PROSITE database pattern syntax rules (http://prosite.expasy.org/). The motif includes single-letter amino acid residues, or a single symbol represents the physical properties of the residues (Table S2); for example, RGD is the abbreviation for Arg–Gly–Asp and PPxY for Pro–Pro–any amino acid–Tyr.

An additional module is implemented for editing phylogenetic tree files. In many cases, the sequences’ headers are long or contain special characters, which may cause errors when these sequences are parsed by sequence alignment or phylogenetic tree reconstruction tools. This option allows changing full headers to shorter accession numbers. The species names (binominal nomenclature) and the accession numbers can be presented in the final phylogenetic tree (Figs S6 and S7), which eases visualization of the final phylogenetic trees.

Another module is implemented to build a universal consensus or molecular signature for multiple sequences using IUPAC rules (Tables S3 and S4). This consensus can be generated for either proteins or genes. The method implemented in the tool takes into account the physical characteristics of the multiple residues within the particular position in protein sequences. The input file for this function is a multiple aligned fasta file. For nucleic acids, if the bases in a position are homogeneous (conserved), a single-letter base is retained in the consensus; otherwise the bases follow the IUPAC-IUB nomenclature system (Table S3) (Sobhy & Colson, 2012). For proteins, the conserved residues are written to consensus. The heterogeneous residues can be (i) bracketed (e.g. [FHWY]A[ED]CT[HYT]) or (ii) represented by a single-letter abbreviation [e.g. aA-CTx; where ‘a’ denotes aromatic residues (F, H, W or Y), ‘-’ denotes negative/acidic residues (E or D) and ‘x’ denotes residues that do not share common properties] (Table S4).

Shetti is a portable and standalone program. It is developed in C#.NET and runs on Windows platforms (Vista/7/8) without preliminary installations; Microsoft.NET Framework is required for older versions. Shetti is free to use for academic and research purposes. Using a PC with 4 GB of RAM, Shetti can load more than 15 000 sequences to memory and present them in an ordered list or table within 10 s. The program's user guide provides a detailed method and a case study.

## Conclusion

Shetti is a simple, user-friendly, robust and fast tool, which integrates several features to manipulate multiple long sequences, and search for particular information within the sequences. This makes Shetti a powerful tool that meets the needs of biologists and microbiologists without prior knowledge of bioinformatics.

## Supplementary Data

623 Supplementary Data

## References

[mgen000035-AnzaldiAnzaldi1] Anzaldi L. J. Muñoz-Fernández D. Erill I. (2012). BioWord: a sequence manipulation suite for Microsoft Word BMC Bioinformatics 13 124 10.1186/1471-2105-13-124 . 22676326 PMC3546851

[mgen000035-Benson1] Benson D. A. Cavanaugh M. Clark K. Karsch-Mizrachi I. Lipman D. J. Ostell J. Sayers E. W. (2013). GenBank Nucleic Acids Res 41 (D1), D36–D42 10.1093/nar/gks1195 . 27899564 PMC5210553

[mgen000035-Mi1] Mi T. Merlin J. C. Deverasetty S. Gryk M. R. Bill T. J. Brooks A. W. Lee L. Y. Rathnayake V. Ross C. A. other authors (2012). Minimotif Miner 3.0: database expansion and significantly improved reduction of false-positive predictions from consensus sequences Nucleic Acids Res 40 (D1), D252–D260 10.1093/nar/gkr1189 . 22146221 PMC3245078

[mgen000035-Okonechnikov1] Okonechnikov K. Golosova O. Fursov M. UGENE team (2012). Unipro UGENE: a unified bioinformatics toolkit Bioinformatics 28 1166–1167 10.1093/bioinformatics/bts091 . 22368248

[mgen000035-Olsen1] Olsen L. R. Kudahl U. J. Simon C. Sun J. Schönbach C. Reinherz E. L. Zhang G. L. Brusic V. (2013). BlockLogo: visualization of peptide and sequence motif conservation J Immunol Methods 400-401 37–44 10.1016/j.jim.2013.08.014 . 24001880 PMC3856553

[mgen000035-Sobhy1] Sobhy H. Colson P. (2012). Gemi: PCR primers prediction from multiple alignments Comp Funct Genomics 2012 783138 10.1155/2012/783138 . 23316117 PMC3535827

[mgen000035-Xia1] Xia X. (2013). DAMBE5: a comprehensive software package for data analysis in molecular biology and evolution Mol Biol Evol 30 1720–1728 10.1093/molbev/mst064 . 23564938 PMC3684854

