# Shetti, a simple tool to parse, manipulate and search large dataset of sequences

**DOI:** 10.1099/mgen.0.000041

**Published:** 2015-11-30

**Authors:** Haitham Sobhy

*MGen*, 2015, **1**. DOI: 10.1099/mgen.0.000035

The Publisher apologizes for the following error in the manuscript, ‘Mati parses UniProt Knowledgebase and NCBI GenBank flat files and visualizes them as a table’ which should be ‘Shetti parses UniProt Knowledgebase and NCBI GenBank flat files and visualizes them as a table’.

