# Supplementary material for: Shetti, a simple tool to parse, manipulate and search large datasets of sequences
Source: Microb Genom. 2015 Nov 6;1(5):e000035. doi: 10.1099/mgen.0.000035 (PMC5320677; doi:10.1099/mgen.0.000035)

Supplementary Information SI-1

Supplementary tables

Table S1. Calculating protein molecular weight.

The equation: **Protein Mol. Wt. = ∑ amino acids Mol. Wt. – (18.015 * (sequence length – 1))**.
Source: http://web.expasy.org/protscale/pscale/Molecularweight.html

Amino acids	Symbols	Symbols	Molecular weight
Alanine	Ala	A	89
Arginine	Arg	R	174
Asparagine	Asn	N	132
Aspartic acid	Asp	D	133
Cysteine	Cys	C	121
Glutamine	Gln	Q	146
Glutamic acid	Glu	E	147
Glycine	Gly	G	75
Histidine	His	H	155
Isoleucine	Ile	I	131
Leucine	Leu	L	131
Lysine	Lys	K	146
Methionine	Met	M	149
Phenylalanine	Phe	F	165
Proline	Pro	P	115
Serine	Ser	S	105
Threonine	Thr	T	119
Tryptophan	Trp	W	204
Tyrosine	Tyr	Y	181
Valine	Val	V	117

Table S2. Single-letter and single-physical properties symbol abbreviations used to search for pattern-motifs.

The user can use amino acid residues directly. The alternative amino acids are bracketed, as in column 2. Some symbols also can be used, column 3. The tool then searches for these residues in sequences. For multiple patterns, they should be separated by “;”. For details, please see the program’s user guide.

Note: The symbols presented in this table are customized for searching purpose only in the tool. In PROSITE “<” and “>” denote N and C-terminals motifs. In Shetti, to terminal search, please use the check-boxes. These symbols or motifs might be changed based on the new literatures.

**Residues**	**Representative Motif**	**Symbol**	**Physical properties**
Arg-Gly-Asp residues	RGD		
Pro-Pro-any amino acid-Tyr	PPxY		
P, and (E or D) residues ≈ PE or PD	P[ED]		
P, but not (E or D) residues	P{ED}		
T is repeated 3 times	PGST(3)		
T is repeated 3 or 5 times	PGST(3,5)		
T is repeated > 3 times	PGST(3,)		
E or D	[ED]	- “hyphen”	Negative - Acidic
H, K or R	[HKR]	+	Positive - Basic
S or T	[ST]	=	Alcohol
C or M	[CM]	*	Sulfur containing
I, V or L	[IVL]	?	Aliphatic
A, G or S	[AGS]	&	Tiny
F, H, W or Y	[FHWY]	@	Aromatic
D, E, H, K, N, Q, R, S or T	[DEHKNQRST]	%	Polar / hydrophilic
A, C, F, G, V, L, I, P, W, M or Y	[ACFGVLIPWMY]	!	Non-Polar / hydrophobic
C, W, N, Q, S, T, Y, K, R, H, D or E	[CWNQSTYKRHDE]	#	H-bond
Any amino acid	Any amino acid	x	No common properties
Multiple patterns	PPxP; PPP; CXXC;<br>CX(6)C; LYPX(1,)L;<br>P[ST]AP; ++X+		

Table S3. Standard single-letter nucleic acid abbreviations used to generate final consensus sequence.

If all the nucleic acid bases in a position are the same (conserved base), a representative symbol is saved in consensus. If the bases are different (divergent bases), the representative IUPAC base is written to the consensus. Details can be found in the program documentation.

Note: The sequence should be in aligned fasta format.

Symbol	Meaning	Base represented	Complement
A	Adenine	A	T or U
C	Cytosine	C	G
G	Guanine	G	C
T or U	Thymine (DNA) or Uracil (RNA)	T or U (RNA)	A
R	puRine	A or G	Y
Y	pYrimidine	C or T	R
W	Weak	A or T	W
S	Strong	C or G	S
M	aMino	A or C	K
K	Keto	G or T	M
V	not T or U (RNA), V comes after T	A, C or G	B
H	not G, H comes after G	A, C or T	D
D	not C, D comes after C	A, G or T	H
B	not A, B comes after A	C, G or T	V
N or X	Unknown or aNy	A, C, G or T	N

Table S4. Single-letter amino acid abbreviations used to generate final consensus sequence.

If all the residues in a position are the same (conserved), a single-letter amino acid is saved in consensus. If the residues are different (divergent), the representative single-letter is written to the consensus. This symbol represents a physical characteristic shared between the residues in this position. Otherwise, x residues, which means any residues that do not share common properties. If the same residues in motif are repeated it can be written as AC(3)T, which denotes ACCCT motif. Details and use case can be found in program documentation.

Note: The sequence should be in aligned fasta format.

**Amino acids in a position**	**Representative symbol** “lower-case letter”	**Physical properties**
Gap "-"	**.** “dot sign”	
E and D	**-** “minus / hyphen”	Negative - Acidic
S and T	**o**	Alcohol
C and M	**s**	Sulfur containing
H, K and R	**+** “plus”	Positive - Basic
I, V and L	**l**	Aliphatic
A, G and S	**u**	Tiny
F, H, W and Y	**a**	Aromatic
D, E, H, K, N, Q, R, S and T	**p**	Polar / hydrophilic
A, C, F, G, V, L, I, P, W, M and Y	**n**	Non-Polar / hydrophobic
C, W, N, Q, S, T, Y, K, R, H, D and E	**h**	H-bond
Any, others	**x**	No common properties

Supplementary Figures

Fig. S1. Screenshot of the “List view” mode screen.

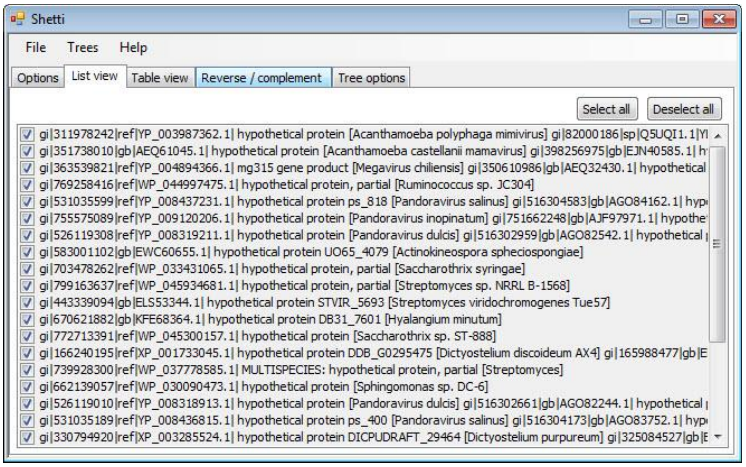

Fig. S2. Screenshot of the “Table view” mode screen.

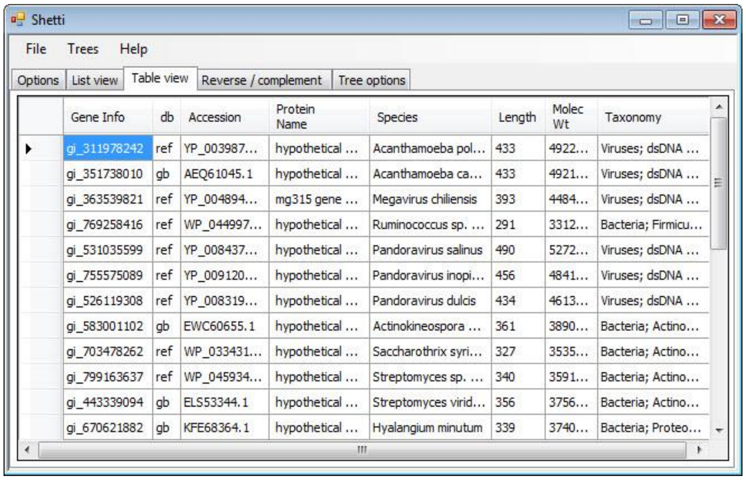

Fig. S3. Screenshot of reverse/complement option in Shetti.

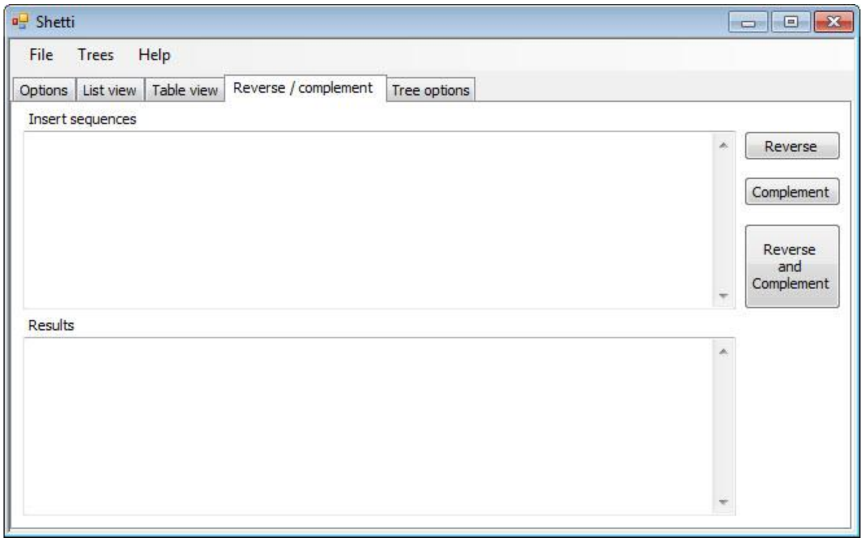

Fig. S4. Screenshot of “Tree options” tab in Shetti.

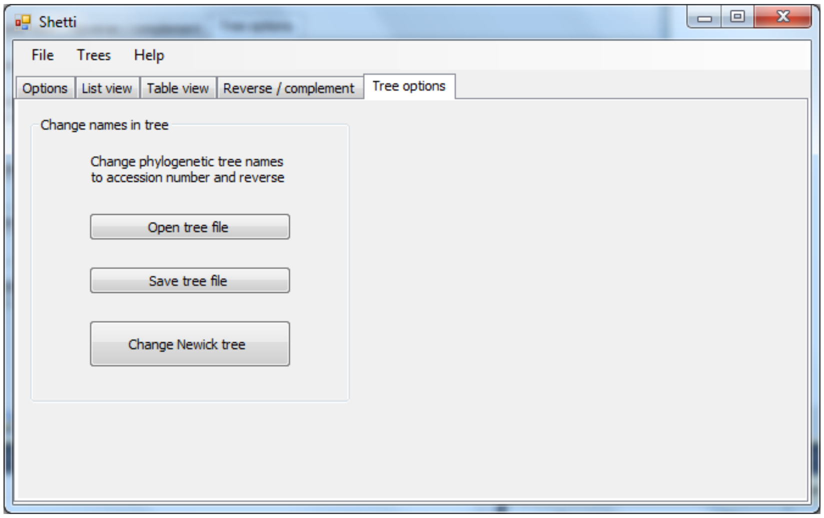

Fig. S5. Shetti accepts files in fasta format as input.

The UniProt protein header format downloaded from UniProt website [No. 1]. The protein and gene coding sequences, and gene feature sequences downloaded from NCBI website [Nos. 2, 3, & 4]. The accession numbers header format saved by Shetti tool [No. 5]. The output of fasta file sequence format resulted from NCBI-BLAST search [No. 6].

The simplest form of fasta format is the following:

> > The first sequence header and description here
> The sequence contains protein amino acid residues or ACGTU nucleic acid bases.....
> > The second sequence header and description here
> The sequence contains protein amino acid residues or ACGTU nucleic acid bases.....

Notes:

Only the **standard fasta formats** mentioned in Fig. S5 can be presented in **Shetti's table view mode**.
These formats can be downloaded from **NCBI** or **UniProt** databases.
The **non-standard fasta headers** should be shown in the **list view**, but **NOT** in the **table view**.

<u>If the fasta file combines standard and non-standard formats, only the standard formats can be viewed in table view.</u>
Some fasta headers are not reviewed by NCBI or UniProt curators, and do not follow the proper format. Thus, these headers are loaded to the memory but cannot be viewed in a table mode. **<u>Instead</u>**, the user can choose to open the headers in **<u>list view, NOT table view</u>**.

<u>The fasta formatted headers are only dedicated for headers visualization mode. Whatever the selected view mode, the sequences will be processed and manipulated in the same manner.</u>
The table and list modes are different ways to visualize the fasta header. They do NOT influence processing of the data. Whatever the view mode you selected to view the fasta headers, the headers and sequences are loaded into memory and parsed in the same manners.

<u>For UniProt Knowledgebase and NCBI GenBank databases **FLAT** file format</u>, further details can be found on the following links: http://web.expasy.org/docs/userman.html and http://www.ncbi.nlm.nih.gov/Sitemap/samplerecord.html, last accessed August 2015.

For details also, please, see the program's documentation and the samples files enclosed in Shetti package compressed file.

1. **UniProt fasta format**

```
>sp|Q5UQH3|53DR_MIMIV Putative 5'(3')-deoxyribonucleotidase R824
OS=Acanthamoeba polyphaga mimivirus GN=MIMI_R824 PE=3 SV=1
MDNCELVGSRIRLGLDMDGVLFDFDSKIMDKFAEMGIQFTDVKEMSSAVEFDKSIKQRHR
EIYHVPGFFANLPPIKGAVNAYKYLKSLTDINNNKIFEIFIVSTPSFRNQTCCIDKINDL
NKYFGPELLEKVFFCRDKTLVNLDILIDDKPEIRGFNGSSECLSDSNSVTNKMSFQHIRF
```

2. **Protein coding sequence**

```
>lcl|AY653733.1_prot_AAV50276.1_1 [gene=MIMI_R1] [protein=replication
origin binding protein] [protein_id=AAV50276.1] [location=193..2580]
MTYVKKYHPTTKYYGIIHGEKYELQDVLFYSFNYSNREEVCPISGTAHKSNGFYVIETSKGYFMKCHSDK
CKNKKAKYLGPADATDMFVKCANQIDQQYLIMKGGIADAPKEPVKDIIINWLSNDKIKTLAVRSPMGTGK
TTMIKKILDHYDNIKKILWISHRQTLSKQIYGSFKNHGFVNYMDQKGNLFEHDRLIIQIDSLKRIFKYDK
```

3. **Gene coding sequence**

```
>lcl|AY653733.1_cds_AAV50276.1_1 [gene=MIMI_R1] [protein=replication
origin binding protein] [protein_id=AAV50276.1] [location=193..2580]
ATGACTTATGTCAAAAAATATCATCCTACCACTAAATATTATGGTATTATTCATGGTGAAAAGTATGAAT
TACAAGACGTTTTATTTTACAGTTTTAATTACTCTAATAGAGAAGAAGTATGTCCAATTAGTGGTACTGC
ACATAAATCCAACGGATTTTATGTAATTGAAACCAGTAAAGGATACTTTATGAAATGCCATTCTGATAAA
```

4. **Gene feature**

```
>lcl|AY653733.1_gene_1 [locus_tag=MIMI_R1] [location=193..2580]
ATGACTTATGTCAAAAAATATCATCCTACCACTAAATATTATGGTATTATTCATGGTGAAAAGTATGAAT
TACAAGACGTTTTATTTTACAGTTTTAATTACTCTAATAGAGAAGAAGTATGTCCAATTAGTGGTACTGC
ACATAAATCCAACGGATTTTATGTAATTGAAACCAGTAAAGGATACTTTATGAAATGCCATTCTGATAAA
```

5. **Accession numbers**

```
>WP_030931902.1
MSTEIFREEFTGGLVTEGPGAPWRLRPVDGLPEGDGVVRGGPDGLVVVPSAVRPGTRRPAFTEPKPGPDG
APLHLRWGAFTTGGTASARGGFTAVPGELLSVTAEMGLRGFGLRSHPYDDVAEATADPRLGAGGLISVDL
ETGMIFDFFLTHSRLYAVYERLALQPDAAFASFTYCVPVADRTPGTLHRLEVGYDVAAGTAHWKADGQEV
```

6. **NCBI-BLAST output**

```
>gi|311978242|ref|YP_003987362.1| hypothetical protein [Acanthamoeba polyphaga
mimivirus] >gi|82000186|sp|Q5UQI1.1|YL829_MIMIV RecName: Full=Uncharacterized
protein L829 [Acanthamoeba polyphaga mimivirus] >gi|55417438|gb|AAV51088.1|
unknown [Acanthamoeba polyphaga mimivirus] >gi|308204624|gb|ADO18425.1|
hypothetical protein [Acanthamoeba polyphaga mimivirus]
>gi|823719903|gb|AKI81506.1| hypothetical protein [Acanthamoeba polyphaga
mimivirus]
MSNRFDSKPKCRCVAKIDDNYENNCQSKYISKCEIPRNICQRKNIDFFYDFRLKYSDADFDYVFGNDGVVTQNFTGLTVN
SVPFTQTVPIGNEHPKWLKFYKDAFPLYNDREVIFETEMSGVQVIDGNSIPEKMKPRIRNVDDDLRLASGALNVIDPNTW
MVFDFFVTNTAIYAFYERLPFGKTSSTPSNTTSQFGNKSFHDKFTHNGSIHNGSIHNGSIHNGSHCNPNPDVPTDLGNYA
```

Fig. S6. The Newick tree format can be parsed by Shetti.

The simplest tree format is “(A:0.1,B:0.2);”. This raw format can be then parsed by a tree visualization tool to present the tree (Fig. S7).

```
((((WP_030931902.1:0.05252,CAA41639.1:0.05940):0.21986,((EMD2615
8.1:0.00582,WP_039918359.1:-
0.00203):0.23620,WP_019070481.1:0.25056):0.04214):0.03200,((ELS5
3344.1:0.18906,WP_045934681.1:0.17592):0.01449,WP_045300157.1:0.
20344):0.05801):0.02216,((((WP_033431065.1:0.17623,EWC60655.1:0.
21941):0.03085,WP_037778585.1:0.23970):0.02296,WP_030090473.1:0.
30029):0.00964,KFE68364.1:0.27866):0.00417,(EJT52281.1:0.30925,(
((((YP_008319211.1:0.05516,YP_009120206.1:0.06059):0.02227,YP_00
8437231.1:0.07475):0.26994,((WP_044997475.1:-
0.00478,YP_004894366.1:0.03228):0.14928,(AEQ61045.1:0.00088,YP_0
03987362.1:0.00143):0.16890):0.19387):0.01874,(((YP_009120545.1:
0.01215,YP_008436815.1:0.01179):0.00976,YP_008318913.1:0.02239):
0.41074,XP_001697439.1:0.41932):0.00890):0.01362,(XP_001733045.1
:0.35843,XP_003285524.1:0.36509):0.06896):0.03051):0.01243);
```

Fig. S7. The visualization of the final phylogenetic tree.

The tree branches could be labelled with accession numbers only (A), or name of the species and the accession numbers (B).

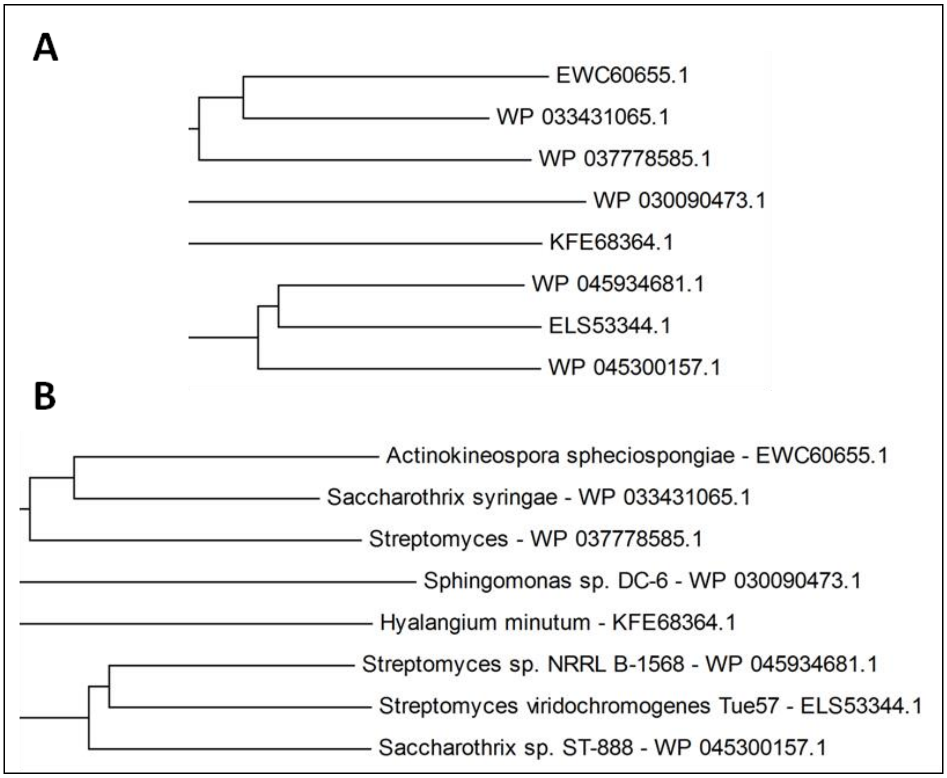

Supplement: Supplementary file 1 — Supplementary Data [file mgen-01-35-s001.pdf]
